# College Note

**Published:** 1903-05

**Authors:** 


### COLLEGE NOTE.

THE medical department of the University of Buffalo will hold its 57th annual commencement, Wednesday, May 6, 1903, at the Teck Theatre, when the degree of doctor of medicine will be conferred upon 49 candidates.

The alumni association will hold its annual meeting on the same day, in the morning of which the classes of '53, '63, '73, '83 and '93 will hold reunions. At the afternoon session, Dr. William T. Sedgwick, professor of sanitary science in the Massachusetts Institute of Technology, Boston, will present the subject of Protection of the public health by the filtration of water supplies, illustrated by the stereopticon. A discussion thereon will be participated in by Drs. Walter D. Greene, Ernest Wende, Herbert M. Hill, Henry R. Hopkins and Herbert U. Williams, of Buffalo; Dr. A. W. Henchell, of Rochester, and Dr. W. R. Campbell, of Niagara Falls. At the banquet, in the evening, which will be given at the Iroquois, at 6.30 o'clock, the principle speaker will be Professor F. D. Fosdick, principal of Masten Park High School, Buffalo. Besides this one there will be other speeches and specialties that will round up the occasion in an entertaining manner.

